# High Incidence of Congenital Syphilis after Implementation of the Brazilian Ministry of Health Ordinances Related to Maternal Diagnostics

**DOI:** 10.3390/pathogens10050606

**Published:** 2021-05-15

**Authors:** Ítala Santos Veras, Caroline Alves Feitosa, Amâncio José de Souza, Leila Carvalho Campos, Galileu Barbosa Costa, Viviane Matos Ferreira

**Affiliations:** 1 Escola Bahiana de Medicina e Saúde Pública, Salvador, Bahia 40290-000, Brazil; italasveras@gmail.com (Í.S.V.); feitosacaroline@gmail.com (C.A.F.); amancio.souza@gmail.com (A.J.d.S.); 2 Laboratório de Patologia e Biologia Molecular, Instituto Gonçalo Moniz, Fiocruz, Bahia 40296-710, Brazil; leila.campos@fiocruz.br; 3 Departamento de Análise em Saúde e Vigilância de Doenças não Transmissíveis, Secretaria de Vigilância em Saúde, Ministério da Saúde, Brasília, Federal District 70723-040, Brazil

**Keywords:** congenital syphilis, incidence, diagnostics, mother-to-child transmission, public health, surveillance, Ministry of Health

## Abstract

The increasing rates of maternal and congenital syphilis (CS) infections are public health concerns and need further investigation in order to provide better assistance in epidemiological surveillance and new strategies for the assistance and prevention of CS. In December 2011, the Brazilian Ministry of Health (BMH) implemented ordinance number 3.242, reinforced in 2012 by ordinance number 77, aiming to improve the quality of the syphilis diagnosis system using rapid tests. Here, we evaluate the incidence, lethality, and possible factors associated with CS in Salvador, Bahia, in the pre-resolution period (2007 to 2011) and post-resolution (2012 to 2016). An observational, ecological time-series study is conducted using secondary data collected from the National Notifiable Diseases Information System (SINAN). Linear regression analysis to estimate increases or reductions in the mean incidence over time is also performed. A total of 5470 CS cases are analyzed. The incidence ranges from 2.1 cases per 1000 live births in 2007 to 17.1 cases per 1000 live births in 2019, showing a progressive increase in incidence over the years and reduction of lethality in the post-resolution period. The number of CS cases reported prior to the implementation of the ordinances (2007–2011) does not reveal a significant increase in the incidence. However, in the post-ordinances period (2012–2019), there is an average increase of the number of CS cases by three times over the years, with an average increase of 1.8 new cases annually. Our findings highlight the importance of diagnosis and support information in strategies for CS prevention. Furthermore, these data show a positive impact of resolutions on the diagnosis and evolution of the disease.

## 1. Introduction

Mother-to-child transmission of syphilis has been considered a global public health burden since the 15th century, and approximately 1.5% of pregnant women (more than two million) develop clinical syphilis every year worldwide [1,2]. The absence of adequate treatment of pregnant women diagnosed with syphilis can cause spontaneous abortion, stillbirth, and early infant death; about 80% of affected pregnancies result in adverse outcomes [3,4].

Congenital syphilis (CS) is a consequence of the hematogenous dissemination of Treponema pallidum through the placenta from infected untreated or inappropriately treated pregnant women [5]. In the early stages of the disease, pregnant women are more prone to CS due to a high concentration of bacteria in the body; these may be transmitted through contact with the newborn via genital lesions in the birth canal [6]. The most common symptoms associated with CS are described as early congenital syphilis, with the presence of cutaneous or mucosal lesions, bone lesions, nervous system lesions, and respiratory changes, and late congenital syphilis, characterized by neurological deafness, interstitial keratitis, Hutchinson’s teeth, and developmental disabilities [7,8].

Aiming to prevent transmission in pregnant women, the World Health Organization (WHO) established in 2007 strategies for CS elimination, which include increasing access to and the quality of maternal medicines and health services, treatment of pregnant women and their partners, establishment of surveillance, and monitoring and evaluation of health systems [3].

Recently, Brazil has experienced a period of increase in syphilis cases, which was declared a public health emergency in 2016 [9]. Between 2010 and 2018, 479,730 cases of acquired syphilis were reported, the majority affecting women (60.1%), with an increase of 31.8% in the detection rate between 2016 and 2017 [10]. In December 2011, the Brazilian Ministry of Health (BMH) implemented ordinance number 3.242, reinforced in 2012 by ordinance number 77, aiming to improve the quality of the syphilis diagnosis system using rapid tests [11,12]. However, since the implementation of the ordinances in 2012, epidemiological profile reports on syphilis have been poorly explored. In the present study, we aim to describe the incidence, lethality, and factors associated with CS cases in Salvador, Brazil, before (2007–2011) and after (2012–2019) the implementation of BMH ordinances numbers 3.242 and 77, and explore the epidemiological scenario.

## 2. Results

**Figure 1:**
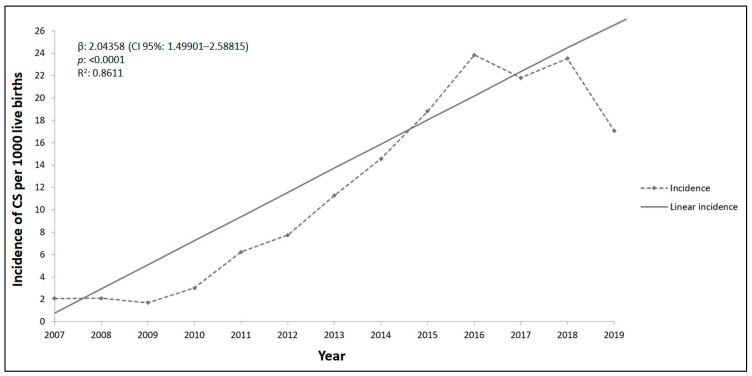
Rates and linear regression as a function of time of incidence of congenital syphilis in Salvador city, State of Bahia, from 2007 to 2019. β indicates the average of increase rate of syphilis annually; p value indicates the statistical significance; R^2^ indicates the variation present in the data.

A total of 5470 cases of CS were reported in Salvador during 2007–2019. The incidence ranged from 2.1 cases per 1000 live births in 2007 to 17.1 cases per 1000 live births in 2019, with an average increase of 2.04 new cases (β = 2.04, 95% CI: 1.49–2.59, *p* < 0.001) annually (Figure 1).

**Figure 2:**
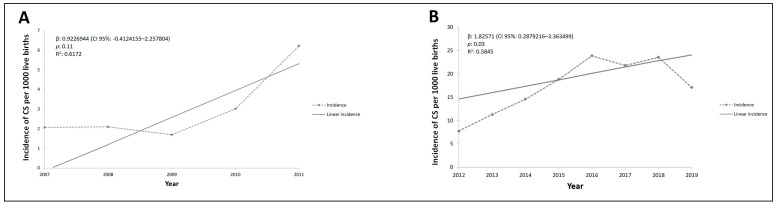
Rates and linear regression as a function of time of incidence of congenital syphilis in Salvador city, State of Bahia, during the pre-resolution period (**A**), and during the post-resolution period (**B**). β indicates the average of increase rate of syphilis annually; *p* value indicates the statistical significance; R^2^ indicates the variation present in the data.

The number of CS cases reported prior to the implementation of the ordinances (2007–2011) did not reveal a significant increase in incidence (β = 0.92, 95% CI: [−0.41]–2.25, *p* = 0.11) (Figure 2A), although a proportional increase of 1.8 times was found at the end of this period. In the post-ordinances period (2012–2019), there was an average increase of the number of CS cases by three times over the years, with an average increase of 1.8 new cases annually (β = 1.8, 95% CI:0.28–3.36, *p* = 0.03, R² = 99.5%) (Figure 2B).

The cases were more frequent in women than in men (Table 1). Furthermore, individuals who self-reported mixed skin color experienced an increase of 7.3% in incidence from the pre- (2007–2011) to the post-ordinances period (2012–2019), while there was an increase of up to 16.3% of CS in mothers with a higher educational level (*p* = 0.004) (Table 1).

**Figure 3:**
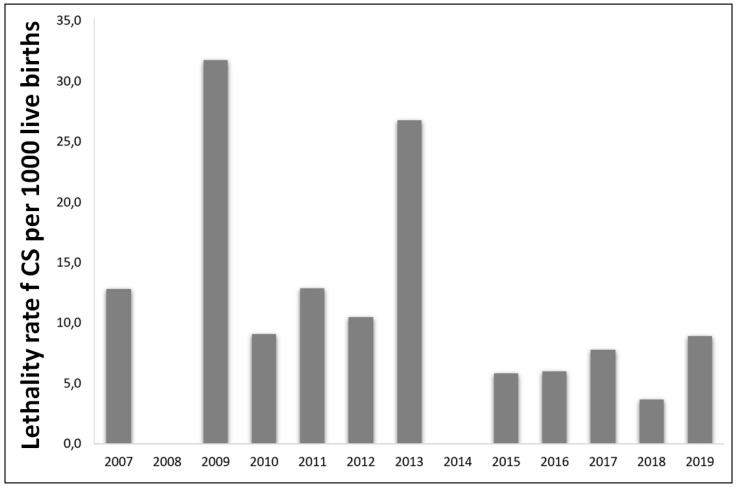
Lethality rates of congenital syphilis in children <1 year of age per 1000 live births, Salvador, Brazil, from 2007 to 2019.

During the 2012–2019 period, there was an increase in the cases of CS where the mothers attended prenatal care (30.6%; *p* < 0.0001), with an increase in the number of maternal syphilis diagnoses during prenatal care (61.9%; *p* < 0.0001), and a decrease in the number of postpartum cases (59.0%; *p* < 0.0001) (Table 2). Additionally, we observed an increase of 91.8% in the percentage of treated partners (*p* < 0.0001). Interestingly, there was a slight decrease in the abortion (0.1%) and stillbirth rates (0.04%) (Table 2). On the other hand, the lethality rate reached a peak in 2009 (31.7%), dropped in the following years, and increased again in 2013 (26.8%), presenting a range of 5.8–8.9% during the 2015–2019 period (Figure 3).

## 3. Discussion

Our study describes the epidemiological profile of CS before and after the implementation of the Brazilian Ministry of Health ordinances related to maternal diagnostics. The three-times increase in CS incidence during the post-resolution period could be explained by the increased access to the diagnosis of syphilis with the use of rapid tests in basic healthcare units, according to ordinances no. 3.242 and 77 [11,12]. Our findings also show a high incidence of CS compared to other regions of Brazil during the same period [13]. It is important to mention that this evidence does not meet the WHO criteria for CS elimination that aims to reach an incidence of less than 50/100,000 live births [3]. The post-resolution period was marked by a predominance of affected female and brown skin color newborns, corroborating previous studies [14,15]. Although other studies reported a higher incidence in mothers with a low level of education [16], our findings have demonstrated an opposite correlation, which could be associated with better access to prenatal care. This finding draws attention to the lack of (or possibly neglected) access to health services, which could create challenges to public health.

With the expansion of the diagnosis of syphilis and the adherence to using the rapid tests after the proposed resolutions, along with the formation of the stork network [17], there was an increase in the number of pregnant women who attended antenatal care, as documented in other regions of Brazil [18,19,20,21,22,23], thus favoring a greater detection of CS cases. Additionally, it was possible to observe a reduction of cases where diagnosis was not performed, and cases of diagnosis performed after childbirth [14,20]. This fact was also exposed by Swartzendruber and colleagues, who reported an increase in antenatal coverage after the adoption of rapid tests for syphilis [24]. Additionally, a study carried out in Mozambique demonstrated greater accuracy of the rapid test when compared to the non-treponemal test [25]. Thus, the use of the rapid test in an environment with limited resources is a useful strategy since they are easy to use and quick to provide results, with readings back after between 8 and 20 minutes [26].

Our study showed reductions in abortion, stillbirth, and mortality rates after an improvement of CS diagnosis. Furthermore, there was an increase in the proportion of treated partners. This fact could also be associated with the previous low number of men attending health services and the national shortage of benzathine penicillin G since 2014, considered the first-choice treatment for syphilis [27]. The prior lack of treatment of partners could have caused higher possibilities of mothers’ reinfection. Additionally, the absence and/or inadequacy of the mother’s treatment still needs to be considered.

It must be noted that our study has limitations. We used secondary data that could potentially be incomplete, possibly affecting our results. Furthermore, the underreporting of data could underestimate the real incidence of CS, indicating that improvements are needed during the completion of reporting forms.

Although several studies have shown an increase in the percentage of ignored/white fields [22,26,27] over the years, a significant decrease of ignored fields was observed in most of the variables used in our study. The reduction of ignored cases and increase in discarded cases may indicate an improvement in the completion of reporting forms, caused by improving detection of syphilis due to the resolutions.

## 4. Materials and Methods

### 4.1. Study Design and Population

An observational, ecological time-series study was conducted using secondary data collected from the National Notifiable Diseases Information System (SINAN) in Salvador, Bahia State (northeast region of Brazil). Data was collected through the health information Generic Public Domain Tab (TABNET) contained on the website of the Secretaria do Estado de Saúde da Bahia (SESAB), Ministry of Health, Brazil (http://tabnet.datasus.gov.br accessed on 3 August 2020). Confirmed cases of CS reported before and after the implementation of ordinances no. 3.242 and no. 77 were recorded. Confirmed cases outside the chosen period, cases from other states and cities, and cases where information about the city of residence was not available on SESAB’s TABNET platform were excluded.

### 4.2. Data Analysis

Incidence rates were calculated by dividing the number of cases for the total of live births during a given period (adjusted to a 1:1000 ratio), while the infant mortality rate was calculated by dividing the number of deaths due to syphilis by the total number of cases. The lethality and risk factors associated with congenital syphilis were analyzed according to the frequency of newborns’ gender, ethnicity, and age; mother’s education and age; partner’s treatment status; and the presence of maternal syphilis, using the chi-squared test and Fisher’s exact test when appropriate. Linear regression analysis was performed using STATA software version 15 (STATA Corp., College Station, TX, USA) to estimate increases or reductions in the mean incidence over time, with 95% confidence intervals, considering a level of significance of 0.05. Population estimate information was obtained for the State of Bahia from the Brazilian Institute of Geography and Statistics (IBGE) and used the 2010 national census [28].

### 4.3. Ethical Considerations

In this study, secondary data were collected from a public domain database (SINAN); therefore, no direct consent from study participants was needed, nor submission to a Research Ethics Committee.

## 5. Conclusions

In conclusion, the implementation of the BMH ordinances had a positive impact on the diagnosis of maternal syphilis, adherence of pregnant women to attending antenatal care, and reductions in the lethality rates of CS. However, CS continues to have a high incidence rate, which points to the need to improve public health policies, including health education and awareness programs and the integration of partners in follow-up prenatal care and treatment. Furthermore, it is essential to improve the training of healthcare professionals, emphasizing the importance of correctly filling out notification forms to better assist in epidemiological surveillance, thus enabling new strategies for the assistance and prevention of CS.

## Figures and Tables

**Table 1:** Demographic characteristics of confirmed congenital syphilis cases before and after implementation of the Brazilian Ministry of Health ordinances no. 3.242 and 77, 2007–2019.

Demographics	Total Cases *	Pre-Resolution Era (2007–2011) n (%) **	Post-Resolution Era (2012–2019) n (%) **	*p* Value ^†^
Newborns’ gender	5470	444	4481	
Female	2540	211 (47.5)	2329 (52.0)	0.07
Male	2385	233 (52.5)	2152 (48.0)	
Newborns’ age	5469	556	4830	
≤ 6 days of life	5228	534 (96.0)	4694 (97.2)	0.09
≥ 7 days of life	153	22 (4.0)	131 (2.7)	
Newborns’ skin color	3631	254	3377	
Non-black and/or non-mixed	159	14 (5.5)	145 (4.3)	0.07
Black	495	49 (19.3)	446 (13.2)	
Mixed	2977	191 (75.2)	2786 (82.5)	
Mother’s education level	3045	287	2758	
Never studied	39	12 (4.2)	27 (1.0)	0.004
≤ 5 years	1757	203 (70.7)	1554 (56.3)	
≥ 6 years	1249	72 (25.1)	1177 (42.7)	

* Totals may not add up to 100% due to missing information. ** % values were calculated by column. ^†^ *p* value was calculated using chi-squared test.

**Table 2:** Factors related to confirmed congenital syphilis cases before and after implementation of the Brazilian Ministry of Health ordinances no. 3.242 and 77, 2007–2019.

Factors	Total Cases * n (%)	Pre-Resolution Era (2007–2011) n (%) **	Post-Resolution Era (2012–2019) n (%) **	*p* Value
Attended prenatal care	4434	396	4038	
Yes	3537	247 (62.4)	3290 (81.5)	<0.0001 ^†^
No	897	149 (37.6)	748 (18.5)	
Mothers’ diagnosis	4811	441	4370	
Prenatal	2623	154 (34.9)	2469 (56.5)	<0.0001 ^†^
During delivery	1723	193 (43.7)	1530 (35.0)	
After delivery	431	85 (19.3)	346 (7.9)	
Non-realized	34	9 (2.0)	25 (0.6)	
Final diagnosis	5024	488	4536	
Early CS	3945	404 (82.8)	3541 (78.0)	<0.0001 ^‡^
Late CS	8	2 (0.4)	6 (0.1)	
Abortion	9	2 (0.4)	7 (0.1)	
Stillbirth	68	35 (7.2)	33 (0.7)	
Inconclusive	994	45 (9.2)	949 (20.9)	
Newborns’ CS outcome	4596	434	4162	
Alive	4528	424 (97.7)	4104 (98.6)	0.13 ^†^
Died due to syphilis	44	7 (1.6)	37 (0.9)	
Died from other causes	24	3 (0.7)	21 (0.5)	
Partners’ treatment	4236	331	3807	
Yes	2646	114 (34.4)	2512 (66.0)	<0.0001 ^†^
No	1590	217 (65.6)	1295 (34.0)	

* Totals may not add up to 100% due to missing information. ** % values were calculated by column. ^†^ *p* value was calculated using chi-squared test. ^‡^ *p* value was calculated using Fisher’s exact test.

## Data Availability

Not applicable.
